# Exogenous Abscisic Acid Alleviates Harmful Effect of Salt and Alkali Stresses on Wheat Seedlings

**DOI:** 10.3390/ijerph17113770

**Published:** 2020-05-26

**Authors:** Xiaoyu Li, Shuxin Li, Jinghong Wang, Jixiang Lin

**Affiliations:** 1 Key Laboratory of Wetland Ecology and Environment, Northeast Institute of Geography and Agroecology, Chinese Academy of Sciences, Changchun 130102, China; lixiaoyu@iga.ac.cn (X.L.); lsx@nefu.edu.cn (S.L.); 2 College of Landscape Architecture, Northeast Forestry University, Harbin 150040, China; yuanlin@nefu.edu.cn

**Keywords:** abscisic acid, alkali stress, biomass, inorganic ions, organic solutes

## Abstract

Exogenous hormones play an important role in plant growth regulation and stress tolerance. However, little is known about the effect of exogenous abscisic acid (ABA) on wheat seedlings under salt and alkali stresses. Here, a pot experiment of saline and alkaline stresses (0 and 100 mmol/L) in which ABA water solution (0, 50 and 100 μmol/L) was sprayed on wheat seedlings was conducted to study the alleviative effectiveness of ABA on salt and alkali stresses. After spraying ABA (50 μmol·L^−1^), shoot biomass increased 19.0% and 26.7%, respectively. The Na^+^ content in shoots reduced from 15-fold and 61.5-fold to 10-fold and 37.3-fold in salt and alkali stresses, compared to controls. In addition, proline and organic acid synthesis in shoots also reduced significantly, but the soluble sugar content increased under alkali stress. A high concentration of ABA (100 μmol·L^−1^) had no significant effects on biomass and ion content in wheat seedlings under both stresses. In conclusion, foliar application of ABA with moderate concentration could effectively accelerate shoot growth of salt-induced wheat seedlings by adjusting the levels of ions and organic solutes.

## 1. Introduction

Salinity is a major environmental stress in arid and semi-arid regions of the world, which drastically decreases crop value and productivity [1]. Soil salinization and alkalization often occur simultaneously throughout the world. For example, alkalinized meadow covers more than 70% of the land area and is still expanding in the northeast of China [2]. Yang et al. (2007) have indicated that alkaline salts (e.g., NaHCO_3_ and Na_2_CO_3_) in the soil were more harmful to plants than neutral salts (e.g., NaCl and Na_2_SO_4_), which added pH stress besides osmotic stress and ion toxicity [3]. Therefore, it is very important to improve salt-alkali tolerance of plants in salt-alkaline soil.

Salinity or high pH can retard plant growth and development, and some methods were brought up for enhancing alkali tolerance. Up to now, these methods included gypsum [4], K [5], Zn [6], arbuscular mycorrhizal biofertilizer [7] and grafting [8,9] supplements, and most of these showed significant amelioration effects on the fruits or vegetables. Few reports researched salt-alkali tolerance of crops, especially under alkaline condition. The physiological responses of crops in the alkaline soil and the improvement of their alkaline tolerance are still unclear.

As plant growth regulators, plant hormones are active members of the signal cascade, which induces stress responses [10]. For higher plants, abscisic acid (ABA) is well known as a stress hormone capable of inducing several adaptations for coping with abiotic environmental stress, such as salt, desiccation, low-temperature and osmotic stresses [11,12]. It promotes stomatal closure by rapidly altering ion fluxes in the guard cells and involves modifications of gene expression [13]. When plants are grown under stress conditions, ABA levels increase within a few minutes to several hours, depending on the type and severity of the stress [14,15,16]. The acclimation of plants to salt stress is regulated by the ABA signaling pathway [17,18]. A pretreatment with ABA has been found to alleviate the inhibitory effect of NaCl on the growth and translocation of assimilates [19,20]. The effect of ABA application on the adaptation and tolerance of plant to alkaline stress was still unknown.

Wheat (*Triticum aestivum*) is an important crop worldwide. Improvement of its salt-alkali tolerance is necessary [21]. There are some reports on the physiological mechanisms of NaCl stress and methods for improving salt tolerance in durum and bread wheat [21,22,23]. A few papers also reported the effect of alkaline stress on growth and photosynthesis in wheat [24,25]. Until now, little is known on the methods for enhancing alkali tolerance in wheat. Therefore, a short-term experiment was conducted to explore the effectiveness of ABA in mitigation of the adverse effects of salinity and alkalinity and also to investigate the possible mechanisms of ABA enhancement of salt-alkali tolerance in the seedlings of wheat.

## 2. Materials and Methods

### 2.1. Plant Materials and Growth Conditions

The pot experiment was conducted at Northeast Normal University in China. The plant material utilized was a salt-tolerant wheat variety, *Triticum aestivum* cv. Jimai 3, developed by Jilin Agricultural University. Its growing period was 82 d and the thousand-seed weight was 37 g. Uniform seeds were sown in 25 cm diameter plastic pots containing washed sand that were out-of-doors and protected from rain. Each pot contained 13 seedlings and was watered with Hoagland’s nutrient solution daily before treatment.

### 2.2. Stress and ABA Treatments

There were two stress types: neutral salt- (NaCl:Na_2_SO_4_ = 9:1) and alkaline salt- (NaHCO_3_:Na_2_CO_3_ = 9:1) nutrient solutions (100 mmol/L). The pH in the salt and alkali stresses were 6.45 and 9.17, respectively. Two levels of ABA water solution, 50 and 100 μmol/L, were used. Control plants were treated with an equivalent amount of nutrient solution with no salt or alkali added and in the absence of ABA. Each treatment was replicated three times.

When the seedlings were 10 days old, control (CK) and stress treatments (S and A) were carried out daily between 17:00–18:00 h with 500 mL treatment solution divided into three portions. ABA (250 mL) was sprayed on shoots on the 1st, 4th, 7th and 10th days during stress treatment. Seedlings were grown for a further 10 days, and the treatment period ended when leaves yellowed and wilted from alkali stress. ABA (Tokyo, Japan Chemical Industry Co., Ltd.) was dissolved in a small amount of ethanol and diluted to the desired concentration with distilled water.

### 2.3. Harvest and Pretreatment

All plants were harvested and washed with tap water followed by distilled water. The shoots and roots were immediately separated and oven-dried for 15 min at 105 °C and then at 70 °C to a constant weight, and the dry weight (DW) was recorded. Fifty milligrams of dried shoot or root tissue was treated with 10 mL deionized water at 100 °C for 1 h, and the extracts were used to determine the levels of inorganic ion, organic acids and soluble sugar. Another 50 mg dry sample was treated with 10 mL of 3% (*w*/*v*) aqueous sulfosalicylic acid, and the extractant was used to determine the proline content.

### 2.4. Ion and Organic Solutes Quantification

An atomic absorption spectrophotometer (TAS-990, Purkinje General, Beijing, China) was used to determine the levels of Na^+^, K^+^, free Ca^2+^ and free Mg^2+^. Quantification of NO_3_^−^, Cl^−^, SO_4_^2−^ and H_2_PO_4_^−^ was performed by ion chromatography (DX-300 ion chromatographic system, DIONEX, Sunnyvale, USA) using an AS4A-SC ion-exchange column, a CDM-II electrical conductivity detector and a mobile phase of Na_2_CO_3_/NaHCO_3_ = 1.7/1.8 mM.

The proline and soluble sugar (SS) contents were estimated spectrophotometrically using ninhydrin [26] and anthrone [27], respectively. The sugar content was calculated from the increase in absorbance at 620 nm on the basis of standard curves obtained with glucose. For the determination of proline, 2 mL of extract was mixed with 2 mL of glacial acetic acid and 4 mL of acid ninhydrin. After 1 h incubation at 100 °C, the tubes were cooled and 4 mL of toluene was added. The absorbance of the upper phase was spectrophotometrically determined at 520 nm. Organic acids (OAs) were measured using ion chromatography (DX-300 ion chromatographic system, DIONEX, Sunnyvale, USA) with an ICE-AS6 ion-exclusion column (20 °C), a CDM-II electrical conductivity detector, a AMMS-ICE II suppressor, a mobile phase with 0.4 mM heptafluorobutyric acid, a flow rate of 1.0 mL min^−1^ and an injection volume of 50 μL. All sample solutions were filtered through a 0.22 μm filter before analysis. The OA content was determined for all types that could be measured in wheat seedlings, including citric, malic, formic, lactic, acetic, succinic and oxalic acids.

### 2.5. Statistical Data Analysis

Data were analyzed using one-way ANOVA implemented in SPSS 13.0 (SPSS Inc, Chicago, IL, USA) between ABA and ABA adding. The normality and homogeneity of variance were tested prior to the ANOVA analysis. A square root transformation was used to meet the statistical requirement when data were not normally distributed. The means and standard errors were reported. Different ABA levels were compared by Least Significant difference (LSD) multiple comparison, and different stress treatments were compared by t-tests (*p* ≤ 0.05).

## 3. Results

### 3.1. Biomass

Growth of wheat seedlings was inhibited by both salt and alkali stresses (Table 1). Reductions in shoots and roots’ dry weight were 38% and 50% under salt stress and 56% and 78% under alkali stress, respectively, compared to the control plants. After ABA application (A + ABA1), the shoots’ dry biomass was improved significantly under alkali stresses, but the value remained lower than those in the control treatments (CK). Although the dry matter of shoots in salt stress increased with ABA application, the increase was not significant (S + ABA1/ABA2). Dry matter of wheat roots was unchanged in both stresses with ABA addition. Shoots’ biomass increased with a low concentration of ABA (50 μmol/L) significantly but did not with high levels (100 μmol/L).

### 3.2. Inorganic Ions

**Figure 1:**
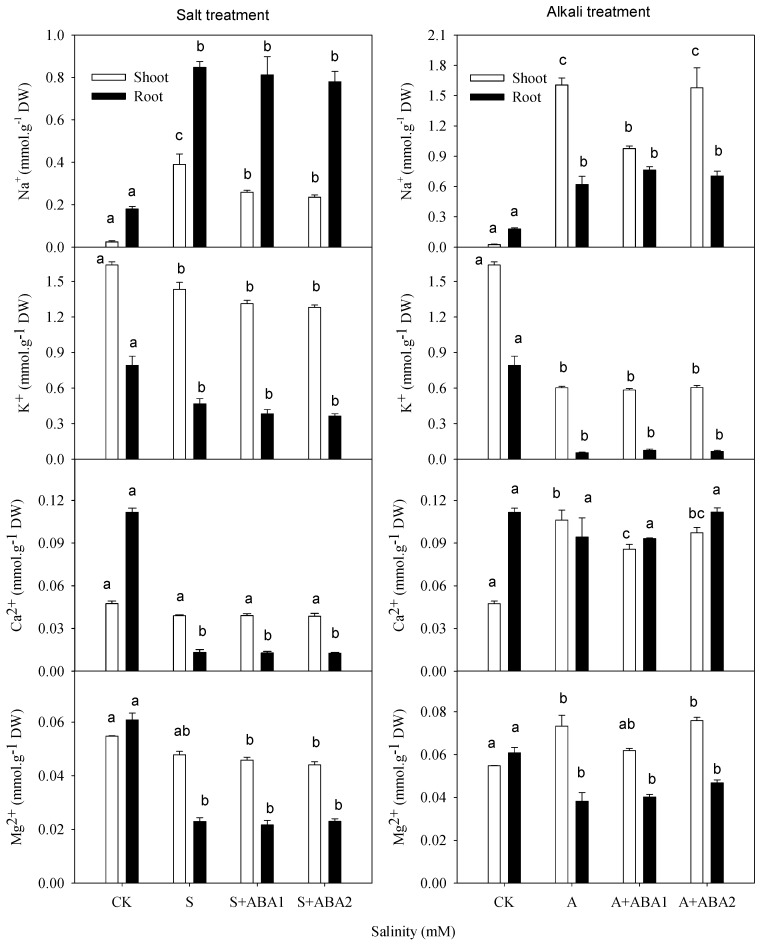
Effect of salt and alkali stress, with or without ABA treatment on the concentration of the main cations in shoots and roots. Means with different letters in columns with different colors are significantly different (*p* < 0.05). DW: dry weight; CK: control, nutrient solution alone; S: 100 mM mixed neutral salts (NaCl:Na_2_SO_4_ = 9:1) added to nutrient solution; A: 100 mM mixed alkaline salts (NaHCO_3_:Na_2_CO_3_ = 9:1) added to nutrient solution; ABA1: 50 μmol ABA; ABA2: 100 μmol ABA. Below was the same.

**Figure 2:**
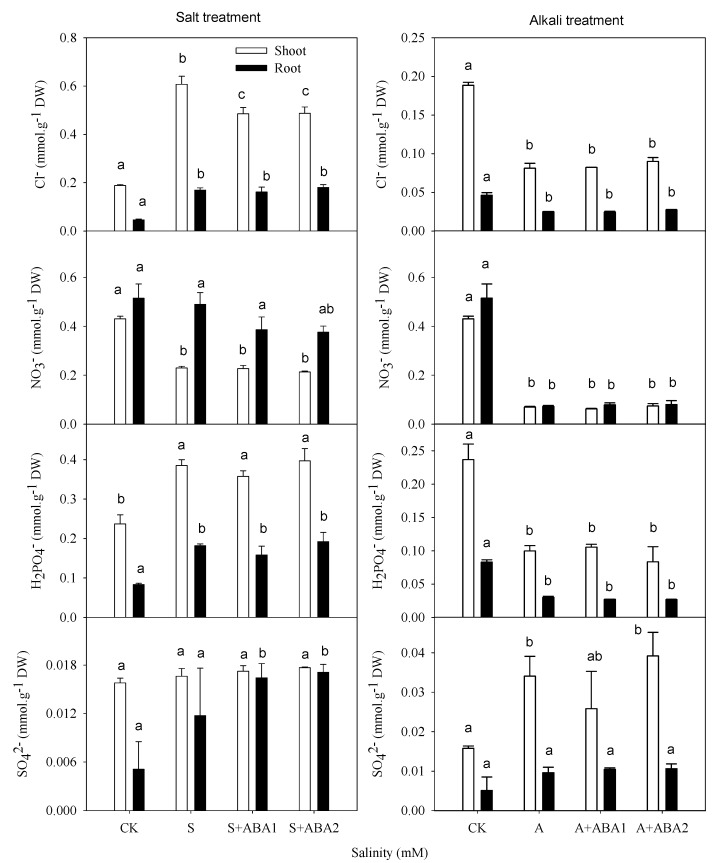
Effect of salt and alkali stress, with or without ABA treatment on the concentration of the main anions in shoots and roots. Means with different letters in columns with different colors are significantly different (*p* < 0.05).

Under salt stress, the Na^+^ content increased and the K^+^ content decreased in both shoots and roots (*p* ≤ 0.05), and the Ca^2+^ and Mg^2+^ only reduced significantly in roots (Figure 1). The Cl^−^ and H_2_PO_4_^−^ contents accumulated markedly in shoots and roots (*p* ≤ 0.05; Figure 2), the NO_3_^−^ content decreased markedly in shoots (*p* ≤ 0.05), and SO_4_^2−^ levels did not change. Under alkali stress, the Na^+^ sharply accumulated, and the K^+^ content decreased in shoots and roots (*p* ≤ 0.01), but the Ca^2+^ and Mg^2+^ contents decreased in roots but increased in shoots (*p* ≤ 0.05; Figure 1). The Cl^−^, NO_3_^−^ and H_2_PO_4_^−^ contents decreased in both shoots and roots (*p* ≤ 0.05; Figure 2), but the SO_4_^2−^ content accumulated in shoots (*p* ≤ 0.05).

**Figure 3:**
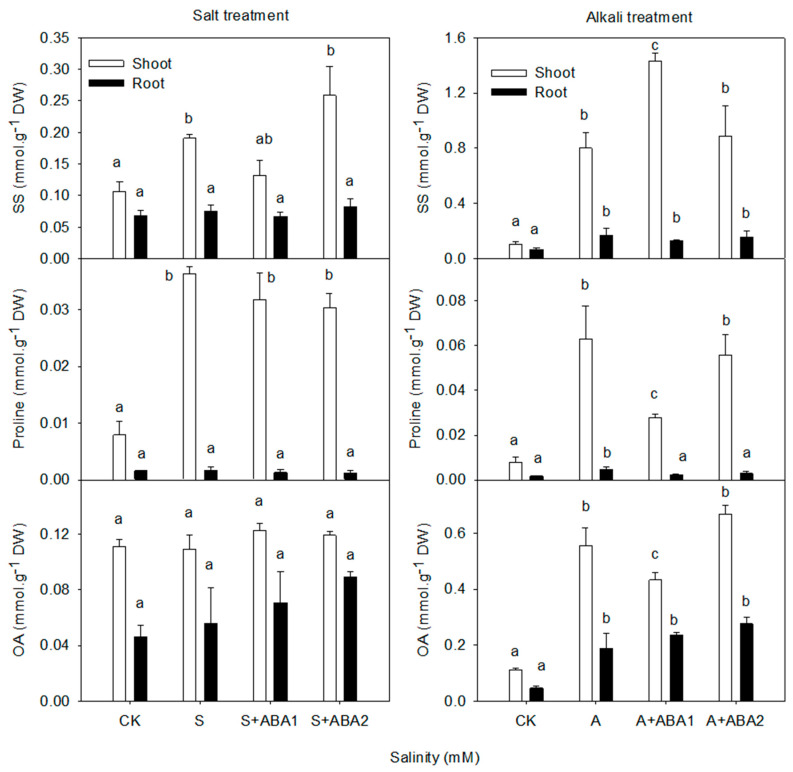
Effect of salt and alkali stress, with or without ABA treatment on the concentration of the main organic solutes in shoots and roots. Means with different letters in columns with different colors are significantly different (*p* < 0.05).

When the seedlings were grown under salt stress, both concentrations of ABA treatment reduced the Na^+^ and Cl^−^ concentrations in shoots (*p* ≤ 0.05; Figure 1 and Figure 2). The ABA treatment decreased the accumulation of Na^+^ from 15-fold to 10-fold relative to the control and decreased the accumulation of Cl^−^ from 3.2-fold to 2.6-fold relative to the control. Under alkali stress, 50 μmol·L^−1^ of ABA significantly decreased the amount of Na^+^ and Ca^2+^ contents in shoots (*p* ≤ 0.01) and decreased Na^+^ from 61.5-fold to 37.3-fold relative to controls, but it did not affect other inorganic ions in the shoots or any ions in the roots (Figure 1, Figure 2 and Figure 3).

### 3.3. Organic Solutes

Salt stress induced wheat accumulated SS and proline but not total OAs significantly (*p* ≤ 0.05; Figure 3). SS and proline increased markedly in shoot but not in roots. The SS, proline and total OAs accumulated significantly in shoots and roots compared to the control (*p* ≤ 0.05; Figure 3) under alkali stress.

After leaf spraying ABA, the SS and proline content did not change obviously under salt stress. However, 50 μmol·L^−1^ of ABA reduced the accumulation of proline and OAs, but it increased the synthesis of SS in shoots. One hundred μmol·L^−1^ ABA did not have effects on shoots. Both levels of ABA had no effects on the organic solutes level of wheat roots.

## 4. Discussion

The dry matter in the seedlings of wheat decreased under salt and alkali stresses. Similar results were also found in the *Kochia sieversiana* [3] and *Lathyrus quinquenervius* [28]. The adverse effect of high pH caused by alkali stress was more serious than salt stress, and the shoots were more strongly inhibited.

Plant hormones play important roles in signal transduction and regulation during plant development, which can also improve the tolerance to environmental stresses. Application of ABA could effectively enhance the leaf growth [29] and induce root changes in morphology and physiology [30] of salt-stressed plants through a potential cellular and signal transduction mechanism. After application of low-level ABA (50 μmol·L^−1^), the dry matter of shoots in both stresses was increased. The increases were 19.0% and 26.7% in salt and alkali stresses, respectively, compared to non-ABA treated seedlings. Hormonal control of cell division and differentiation is clear from the appearance of leaves, which were smaller in area but often thicker [31]. Although the increase in shoots of salt-stressed plants was not significant, the positive effect of ABA adding could be found in physiological changes. The function of ABA depended on the adding regions of plants. For example, ABA elicits different responses depending on the leaf region to promote the resumption of leaf growth [32]. For stress-induced wheat seedlings, the direct region was leaves, so shoot growth was affected but roots did not show significant change with ABA application. The application of 50 μmol·L^−1^ ABA was more effective than the 100 μmol·L^−1^ application.

Na^+^ is one of the main poisonous ions in salt soil. Neither glycophytes nor halophytes can tolerate too much Na^+^ in the cytoplasm. As Na^+^ will enter root cells passively from a saline soil, Na^+^ efflux might increase dramatically, requiring an antiporter such as *SOS1* and a plasma membrane proton pump [33]. High sodic salt level induced the expression of an amiloride-resistant Na^+^/H^+^ antiporter that could account for the remarkable tolerance to NaCl [34]. Parida and Das (2005) and Munns et al. (2008) indicated that one of the mechanisms of salt tolerance was that the root accumulated a lot of toxic ions and prevented transporting into the leaf [13,35]. Some studies reported the ion distribution of wheat seedlings in salinity stress [36]. In the present study, wheat seedlings accumulated more Na^+^ in roots than in shoots (Figure 1). It was consistent with the result of Saqib et al. (2005), which indicated that wheat accumulated Na^+^ mainly in roots and reduced the harmful effect of Na^+^ on the shoots [37]. The high level of Na^+^ and pH in the outer part of the root in alkaline stress resulted in high Na^+^ and H^+^ concentration gradients between intracellular and extracellular root, which made it easier for Na^+^/H^+^ antiporters to export H^+^ and import excess Na^+^. Meanwhile, the high pH condition destroyed the inhibitory ability of wheat transporting Na^+^ to the shoots. Therefore, alkali stress induced more Na^+^ accumulation in the shoots (Figure 1).

Plants usually maintain a lower Na^+^ and higher K^+^/Na^+^ ratio in the cytoplasm under salt stress [38]. Exogenous application of ABA can regulate the pattern of ion accumulation in salt-stressed plants [30]. Our results were consistent with the findings of Etehadnia et al. (2008), who indicated that application of ABA could alter plant responses to salt stress in both resistant and sensitive potato lines [39]. ABA application decreased the Na^+^ content in the shoot, but no change was observed in the root (Figure 1), indicating that exogenous ABA might take part in the gene expression of controlling transport Na^+^ from root to shoot. Khadri et al. (2006, 2007) also showed that the addition of 1 mΜ ABA to a nutrient solution prior to salt stress reduced the negative effects of NaCl, seemingly by limiting sodium translocation to the shoot, and this resulted in the maintenance of a high K^+^/Na^+^ ratio [20,40]. The decrease of Cl^−^ content in salt stress with ABA application corresponded with the change in Na^+^, which was balanced by Cl^−^. These results indicated that the Na^+^ and Cl^−^ toxicity of salt stress was alleviated by applying ABA.

Similar to the function of ABA on salt stress, application of ABA also decreased the Na^+^ content in the shoot under alkali stress. The difference from salt stress was that alkali stress severely restricted the absorption of anions such as NO_3_^−^ and H_2_PO_4_^−^ (Figure 2). The deficit of negative charge and ion imbalance under alkali stress caused plants to accumulate organic acids as organic anions to balance the excess of Na^+^ [3,28]. OA metabolism played a key role in adjusting stable pH, which could decrease the cellular water potential and increase the activity of mechanisms to avoid cell physiological drought [24]. In the present study, added ABA also significantly reduced OA concentration in the shoots of wheat. These results showed that ABA supply ameliorated the pH stress and Na^+^ toxicity resulting from alkali stress.

Proline is widely distributed in higher plants, and it is accumulated in larger amounts than other amino acids in salt-stressed plants [41,42]. Proline acted as a signaling/regulatory molecule to activate multiple responses that were components of adaptation to abiotic stress including salt stress [43]. Some reports argued that the increased proline under stress was a product of but not an adaptive response to stress [44,45]. Exogenous ABA reduced the proline concentration in the shoots and proved that proline was a stress byproduct in alkali-treated wheat. This result was similar to those of Khadri et al. (2006) on the alleviation of salt stress by exogenous abscisic acid in the common bean, which manifested as a decrease in nodule proline concentration [20].

The observed increase in SS may be related to the excess of Na^+^ under alkali stress [46]. Heidari and Mesri (2008) found a positive correlation between Na^+^ concentration and SS, where the ABA supply decreased the accumulation of Na^+^, which could subsequently decrease the SS concentration [47]. However, our experiment actually stimulated the synthesis of SS. According to our data on solutes concentration, SS are the most abundant solute in wheat, and they play an important role in osmotic adjustment. Thus, ABA treatment enhanced the synthesis of SS, which ultimately strengthened the osmotic adjustment and improved the alkali-resistance of wheat.

ABA enhanced the tolerance to alkaline stress by regulating inorganic ion levels and organic solutes synthesis in the shoots of wheat seedlings. In rice seedlings, ABA primes enhanced their tolerance to alkaline stress by upregulating the antioxidant defense system and tolerance-related stress in the roots [48]. Therefore, the growth and physiological responses of seedling organs to alkaline stress were related to the application site of abscisic acid.

## 5. Conclusions

The detrimental effects of alkali stress on wheat seedlings could be alleviated by applying ABA effectively, ameliorating pH stress and Na^+^ toxicity resulting from alkali stress. OAs were the products of pH stress and Na^+^ toxicity, which were reduced by ABA application. In addition, SS synthesis was enhanced, which strengthened the osmotic adjustment and improved seedling alkali-resistance. Under salt stress, Na^+^ and Cl^−^ toxicity was alleviated by applying ABA, which demonstrates that ABA alleviates ionic stress mainly in salt stress. The alleviation of ABA supply was related to the application region on plants. Foliar application of ABA played a direct role in improving shoot growth but not root growth. The positive effect of exogenous ABA was not enhanced with the increasing concentration. A lower concentration (50 μmol·L^−1^) of ABA was more effective than a higher concentration (100 μmol·L^−1^).

## Figures and Tables

**Table 1:** The biomass of wheat seedlings under salt and alkali stresses and abscisic acid (ABA) treatment.

Treatment	Shoots DW (g)	Roots DW (g)	Treatment	Shoots DW (g)	Roots DW (g)
CK	0.341 ± 0.021	0.181 ± 0.006			
S	0.209 ± 0.019	0.086 ± 0.005	A	0.147 ± 0.009 ^b^	0.038 ± 0.004
S + ABA1	0.248 ± 0.020	0.081 ± 0.008	A + ABA1	0.190 ± 0.006 ^a^	0.048 ± 0.002
S + ABA2	0.223 ± 0.011	0.089 ± 0.004	A + ABA2	0.164 ± 0.007 ^b^	0.042 ± 0.001
*P*	0.33	0.61	*P*	0.02 *	0.08

Means with different letters in a column were significantly different (*p* < 0.05). DW: dry weight; CK: control, nutrient solution alone; S: 100 mM mixed neutral salts (NaCl:Na_2_SO_4_ = 9:1) added to nutrient solution; A: 100 mM mixed alkaline salts (NaHCO_3_:Na_2_CO_3_ = 9:1) added to nutrient solution; ABA1: 50 μmol ABA; ABA2: 100 μmol ABA. * indicate significance at *P* < 0.05.

## Abbreviations

ABA	abscisic acid
DW	dry weight
SS	soluble sugar
OA	organic acid
